# Ergometrine for postpartum hemorrhage and associated myocardial ischemia: Two case reports and a review of the literature

**DOI:** 10.1002/ccr3.2516

**Published:** 2019-11-06

**Authors:** Stuart P. E. Spencer, Sandra A. Lowe

**Affiliations:** ^1^ Bankstown‐Lidcombe Hospital Bankstown NSW Australia; ^2^ Royal Hospital for Women Sydney NSW Australia; ^3^ Department of Women's and Children's Health University of New South Wales Sydney NSW Australia

**Keywords:** acute coronary syndrome, anemia, ergometrine, myocardial ischemia, postpartum hemorrhage

## Abstract

Ergometrine is recommended for use in the medical treatment of postpartum hemorrhage. Ergometrine can occasionally precipitate myocardial ischemia in the setting of significant anemia in women without preexisting cardiac risk factors, and it is important to recognize and treat myocardial ischemia in affected patients to prevent severe complications.

## Graphical abstract

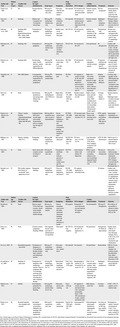

## 1 BACKGROUND

Ergot analogues are commonly administered to women for treatment of postpartum hemorrhage secondary to uterine atony. We describe two cases of women who developed myocardial ischemia due to coronary artery vasospasm following administration of the ergot analogue ergometrine for control of postpartum bleeding and present a literature review of similar cases for comparison. Myocardial ischemia is a rare acute complication of the administration of ergometrine and its analogues in obstetric and gynecologic patients in the absence of preexisting cardiac risk factors. It is important to recognize the rare possibility of inducing myocardial ischemia with ergometrine administration in patients without significant preexisting cardiac risk factors, particularly in the setting of anemia secondary to postpartum hemorrhage.

The ergot analogue ergometrine is recommended by the Royal College of Obstetricians and Gynaecologists as a second‐line pharmaceutical agent for the treatment of uterine atony in cases of postpartum hemorrhage after first‐line oxytocin administration.^1^ In the context of the anemia and hypovolemia that may result from acute postpartum hemorrhage, the administration of ergometrine may rarely precipitate coronary vasospasm and the potential for myocardial ischemia should be recognized as a rare but serious complication^2^, ^3^, ^4^, ^5^, ^6^, ^7^, ^8^, ^9^, ^10^, ^11^, ^12^, ^13^, ^14^ with a mortality rate as high as 18%.^15^ It is important to recognize the potential for myocardial ischemia in patients with no prior risk factors and to correct reversible causes promptly to reduce or prevent morbidity and mortality from myocardial ischemia and infarction. We describe two cases of myocardial ischemia in the context of postpartum hemorrhage and ergometrine administration.

## 2 CASE PRESENTATION

### 2.1 Case one

Patient one was a 48‐year‐old woman at 35‐week gestation (gravida 3 para 2 following in vitro fertilization) who underwent emergency cesarean section for a fourth antepartum hemorrhage of 300 mL after three prior antepartum hemorrhages of unknown etiology between 27 and 35 weeks, without evidence of placenta previa. On examination prior to her surgery, she was hemodynamically stable (heart rate [HR] 76 beats per minute [bpm], blood pressure [BP] 110/70 mm Hg) with a hemoglobin (Hb) of 98 g/L, ferritin of 73 μg/L, and Kleihauer negative. She had been diagnosed with gestational diabetes at 26‐week gestation, successfully controlled with dietary modification alone. She was a nonsmoker with no family history of heart disease and no personal history of dyslipidemia, hypertension, diabetes preceding pregnancy, chronic renal disease, or obesity. She had previously been diagnosed with panic disorder and bulimia nervosa. Her prepregnancy body mass index (BMI) was 22.1 kg/m^2^.

Under spinal anesthetic, a lower‐segment cesarean section was performed, with delivery of a live male infant weighing 2480 g. One hundred micrograms (mcg) of carbetocin was administered at time of delivery. The placenta was delivered piecemeal, and the uterus was exteriorized to place 2 hemostatic sutures to venous sinuses at the placental bed. A total of 500‐mcg ergometrine was administered intraoperatively as per institutional policy for uterine atony in two 125‐mcg intravenous (IV) doses and one 250‐mcg intramuscular (IM) dose. Six mL of prostaglandin F2 alpha was injected into the uterine fundus for ongoing bleeding, and after closure of one uterine layer, a B‐Lynch suture was placed with 0 polydioxanone suture (PDS) to further control uterine bleeding. The second uterine layer was then closed, at which time the patient became anxious and agitated and a decision was made to administer a general anesthetic and intubate the patient. Hemostasis was achieved with closure of the second uterine layer, estimated blood loss for the procedure was 2000 mL, and the patient remained hemodynamically stable throughout the procedure. The postoperative hemoglobin was 67 g/L without ongoing bleeding, and the patient was transfused one unit of packed red blood cells in accordance with institutional policy.

The following morning, 8 hours postoperatively, the patient complained of shortness of breath and a sensation of blockage in her neck and chest without radiation to her back, jaw, or arm. On examination, the HR was 86 bpm, BP was 105/66 mm Hg, and a soft systolic ejection murmur was audible. There were no signs of cardiac failure. Electrocardiogram (ECG) showed sinus rhythm with normal axes and no ischemic changes. Her hemoglobin, 8 hours postoperatively, was 79 g/L. Troponin T was 83 ng/L (normal range < 14 ng/L) on initial review, and 4 hours later, her ECG was unchanged and the patient's troponin T fell to 55 ng/L (Δ−28 ng/L [−33%]). Transthoracic echocardiogram demonstrated normal left ventricular size and function, a mildly dilated left atrium, mild mitral regurgitation with thickened, mobile leaflets, and mild‐to‐moderate tricuspid regurgitation. Type 2 acute myocardial infarction (AMI) was diagnosed, thought to be secondary to anemia and ergometrine administration, with functional mitral regurgitation. A treatment target hemoglobin of 100 g/L was adopted, and the patient was transfused three units of packed red blood cells with a final hemoglobin of 101 g/L being reached 3 days postoperatively. The patient received prophylactic anticoagulation with enoxaparin postoperatively, but did not require any other therapy for treatment of AMI. The symptoms settled spontaneously 24 hours postoperatively, and the patient remained asymptomatic thereafter.

The patient was discharged home on day 15 postdelivery, with the discharge delay being due to her complex psychosocial situation. Three weeks postdelivery, the patient underwent computed tomography (CT) coronary angiography which found no evidence of coronary atherosclerosis and noncontrast CT scanning found her CT coronary artery score to be zero, corresponding to a <1% chance of myocardial infarction over 10 years.^16^

### 2.2 Case two

Patient two was a 35‐year‐old woman, gravida 2 para 1 with a background of a small persistent perimembranous congenital ventricular septal defect (VSD) for which she was asymptomatic. Echocardiogram in her first pregnancy determined that the VSD was of limited clinical significance. She had diet‐controlled gestational diabetes and an otherwise unremarkable antenatal course. Her father had suffered a fatal myocardial infarction at the age of 62, but the patient was a nonsmoker with no history of dyslipidemia, hypertension, preexisting diabetes, chronic renal disease, or obesity.

She presented in spontaneous labor at 39 + 2 weeks gestation. Following a 2‐hour and 1‐minute first stage of labor and a 16‐minute second stage of labor without analgesia, a live male infant weighing 3140 g was delivered vaginally. The patient was administered 10 units of Syntocinon IM with delivery. Despite an estimated postpartum blood loss of 1000 mL, the patient was hemodynamically stable with HR 66 bpm and BP 105/65 mm Hg. The patient was administered an IV infusion of 40 units Syntocinon over 4 hours 250 mcg of ergometrine IV for postpartum hemorrhage. The uterus remained atonic but responded to bimanual palpation and clot evacuation from the vagina and cervix. A further 250 mcg of ergometrine was administered IM. Examination at that time revealed a grade 3C perineal tear, and the patient was taken to theater for perineal repair under spinal anesthesia.

During repair, the patient lost a further 1800 mL of blood secondary to uterine atony and perineal trauma, and the patient was noted to have ST elevation of 2‐3 mm on cardiac monitoring approximately 1 hour after ergometrine administration. This was associated with a complaint of chest tightness, dizziness, and nausea although the HR (80 bpm) and BP (100/40 mm Hg) remained unchanged. One liter of IV crystalloid fluids and noninvasive oxygen supplementation was administered, and cardiac monitoring demonstrated sinus rhythm with normalization of the ST segment. The patient was transfused 3 units of packed red blood cells and administered 1‐g tranexamic acid. Postoperatively, a 12‐lead ECG demonstrated sinus rhythm with ST depression of 1 mm in the inferolateral leads (V4‐6, II, aVF). Investigations at the time demonstrated that the hemoglobin had fallen from 137 g/L on admission to 74 g/L day 1 postoperatively, and serial troponin T testing showed an increase from 7 to 197 ng/L over 6 hours. Chest X‐ray was within normal limits. The patient received enoxaparin 40 mg daily for postoperative thromboembolic prophylaxis but did not require therapeutic anticoagulation or invasive cardiac intervention. The patient was transfused one further unit of packed red blood cells, and on day 2 postoperatively, the Hb had returned to 103 g/L with a troponin T level of 88 ng/L.

Transthoracic echocardiogram on day 3 after delivery demonstrated normal systolic function with no regional wall abnormalities and normal valvular function. The VSD was noted to be present. The patient was discharged home on day 4 of admission. Six weeks postdelivery, the patient underwent stress echocardiography which demonstrated no inducible coronary ischemia.

## 3 DISCUSSION

Ergometrine (ergonovine) is a crystalline alkaloid extract of ergot that is commonly used in obstetric cases to induce tonic uterine contraction through its action on uterine smooth muscle. The amplitude and frequency of uterine contractions and uterine tone are increased; lesser doses see this increased uterine activity interspersed with periods of relaxation, increased doses demonstrate sustained uterine tone without relaxation with a subsequent reduction in uterine blood flow, and the contraction of the uterine wall around bleeding vessels at the placental bed contributes to hemostasis.^17^ Ergometrine also has a vasoconstrictive effect, which can be seen in the disease ergotism whereby damp cereal crops contaminated with ergot‐containing fungus cause progressive vasospasm leading to peripheral ischemia and eventual gangrene.^18^ This vasoconstrictive effect can be used in the provocation of coronary artery vasospasm in cardiac catheterization procedures but has lost favor to acetylcholine, which has a shorter duration of action with less risk of infarction.^9^

Ergometrine is recommended by the Royal College of Obstetricians and Gynaecologists (RCOG) in second‐line pharmacological management of postpartum hemorrhage due to uterine atony^1^ after first‐line administration of oxytocin (Syntocinon). This management approach is supported in the American College of Obstetricians and Gynecologists (ACOG) practice bulletin for postpartum hemorrhage where methylergonovine is recommended as second‐line pharmacological agent^19^ after oxytocin. The Royal Australian and New Zealand College of Obstetricians and Gynaecologists (RANZCOG) also recommends ergometrine as a second‐line pharmacological agent (after oxytocin) in the management of postpartum hemorrhage due to insufficient uterine tone,^20^ but note is made in the guideline of the difficulty in conducting randomized controlled trials for uterotonics in postpartum hemorrhage, and thus, ergometrine has not been well studied in this context.

Ergometrine may be administered IV or IM. The IV route has an immediate onset of uterine contraction but may need to be readministered every 2‐4 hours as necessary, and the patient should be monitored for hypertension, while IM administration has onset of uterine contractions within 2‐5 minutes and is less likely to precipitate hypertension.^21^ Use of Syntometrine (ergometrine‐oxytocin) vs oxytocin alone has been shown to provide a very mild benefit in prophylaxis against primary postpartum hemorrhage >500 mL, but this benefit is not observed with blood loss >1000 mL.^22^

Studies suggest that the incidence of myocardial infarction during pregnancy is between 1 in 16 129 deliveries^23^ and 1 in 35 700 deliveries^24^; however, the mortality rate has been estimated to be as high as 18%.^15^ This high mortality is concerning for a condition that may be rare and difficult to recognize in a patient population that is not traditionally associated with ischemic heart events. Recognizing factors that may predispose myocardial ischemia—such as ergot (including ergometrine and methylergometrine) administration in the setting of hemorrhage, anemia, and the intravascular volume depletion that severe postpartum hemorrhage may precipitate—is crucially important. Anemia may not be recognized as visual estimation of blood loss is liable to underestimate blood loss, and signs of hypovolemic shock are less sensitive in pregnancy.^1^

We conducted a search of the literature in PubMed and MEDLINE using the terms [“ergometrine” or “ergonovine” or “methylergonovine”] and “myocardial ischemia” or “myocardial infarction” or “acute coronary syndrome” and additionally searched references of review articles for cases of ergot‐related myocardial events. This search was performed in November 2016 and repeated in February 2018. Myocardial ischemia in the context of postpartum hemorrhage managed with ergots has been described previously but is very rare.^2^, ^4^, ^6^, ^7^, ^8^, ^9^, ^14^, ^25^, ^26^ In three of the nine cases, the women had no preexisting cardiac risk factors.^6^, ^8^, ^25^ Myocardial ischemia has also been described in three cases when patients were administered oral ergots for induction of abortion,^27^, ^28^ in three patients for routine prophylaxis against hemorrhage following dilatation and curettage^5^, ^13^, ^29^ and in one patient for treatment of abnormal uterine bleeding.^3^ These cases demonstrate that ergot administration is not without the risk of severe side effects even in young women without any objective cardiac risk factors. During resuscitation of a patient with postpartum hemorrhage, consideration should be given to the risk of cardiac injury as a result of both the insult of hemorrhagic shock and administration of ergometrine to control uterine bleeding (see Table 1—Patient Risk Factors and Case Details). The median age for the patients was 34 years old, and 4 of the 17 patients had no documented cardiac risk factors.

**Table 1:** Patient risk factors and case details

Author and Year	Patient age (years)	Cardiac risk factors	Context for ergot administration	Ergot agent	Symptoms	Peak cardiac biomarkers	ECG changes	Cardiac catheterization	Treatment	Outcome
Taylor et al., 1985	22	Nil	Forceps delivery, PPH	200‐mcg IV ergometrine once only	Chest pain with left arm radiation, nausea	Not reported	ST elevation with Q waves in anterior precordial leads	Normal coronary arteries, LV aneurysm	Nil (not recognized)	Aneurysmectomy. Stroke (likely embolic) 1‐y postevent with left hemiparesis
Liao et al., 1991	34	Smoking	Dilation and evacuation for 1st trimester miscarriage	200‐mcg IM methylergometrine once only	Crushing substernal pain and hypotension	Not reported	ST elevation in anterior precordial leads, ST depression II, III, aVF	Normal coronary arteries	Sublingual GTN—symptoms resolved	Diltiazem monotherapy. Patient able to return to previous level of activity
Fukiwara et al., 1993	38	Smoking 20/d Obesity Hypercholesterolemia FHx—father AMI, sister stable angina	Termination of pregnancy	750‐mcg PO methylergometrine for 10 d prior	Sudden onset severe precordial chest pain	CK 376 U/L	ST elevation II, III, aVF	Subtotal occlusion with thrombus of the proximal right coronary artery	Thrombolysis with urokinase	Normal coronary arteries, vasospasm able to be introduced with 0.1‐mg IV methylergometrine
Fukiwara et al., 1993	42	Smoking 15/d	Termination of pregnancy	250‐mcg PO methylergometrine once only	“Oppressive precordial sensation” 4 h after administration	CK 227 U/L	ST elevation II, III, aVF	None performed	SL nitroglycerin	Cardiac catheterization after 4 wks—nil atheroma, coronary vasospasm inducible by placement of catheter at left and right ostia
Roberts et al., 1993	23	Smoking 20/d	Normal pregnancy, breech delivery, routine ergometrine for PPH prophylaxis	500‐mcg IM ergometrine once only	Severe retrosternal chest pain 11 h after delivery	CK 1500 U/L	ST elevation II, III, aVF	Coronary angiogram: normal coronary arteries	IV glyceryl trinitrate and nifedipine, then IV streptokinase	LV angiography showed small area LV dyskinesia. Exercise test 6 wks post showed nil ischemia
Yaegashi et al., 1998	31	FHx AMI (father)	5 d postpartum receiving methylergometrine for uterine atony prophylaxis	750‐mcg PO methylergometrine daily	Substernal pain	CK 3041 U/L	ST‐segment elevation in leads II, III, and aVF ST‐segment depression and reversed T waves in leads I, aVL, V1, V2, and V3	Right coronary artery vasospasm Stenosis in right coronary artery, atrioseptal artery, and posterior descending branch	Heparin, nicorandil, isosorbide, and diltiazem	Coronary angiography on the 30th hospital day showed neither stenosis nor vasospasm of the coronary arteries. Hypokinesis of inferior wall
Nall et al., 1998	28	None	2nd trimester spontaneous miscarriage with dilation and curettage	200‐mcg PO methylergonovine TDS for 6 doses	Right‐sided substernal chest pain	CK 6465 U/L (MB fraction 429.5 ng/mL)	Initially normal, then inferoposterior MI	Right coronary artery and left circumflex artery 100% occluded proximally. Left anterior descending artery patent with proximal ectatic region	Coronary Artery Bypass Grafts x3	Ejection fraction 34% Pathology report revealed coronary artery ectasia secondary to acute and chronic vasculitis
Sutaria et al., Mousa et al., 2000	28	“Heavy” smoker Familial hypercholesterolemia FHx mother and father fatal MI early 40s	Unplanned home birth (short second stage) with routine. Syntometrine prior to completing 3rd stage of labor	500‐mcg IM ergometrine once only (as part of Syntometrine)	“Severe central chest tightness radiating to both arms and associated with profuse sweating, nausea, and breathlessness”	CK 9858 U/L (MB fraction 8%)	ST elevation chest leads with 6‐mm elevation V4, V5	Three‐vessel disease with proximal occlusion LAD	GTN, aspirin, morphine, 3‐mm balloon and 9‐mm NIR stent to occlusion	Anteroseptal hypokinesis on echocardiography
Ribbing et al., 2001	31	Obesity, smoking 30/d 15+ y	Postpartum bleeding	200‐mcg IM methylergometrine once only	Retrosternal chest pain	CK 892 U/L (MB fraction 140 U/L)	ST elevation > 0.3 mV V2‐V6	Large thrombus in proximal LAD, thrombotic complete occlusion LAD periphery	GTN, IV rtPA thrombolysis	Follow‐up at 1 y showed anterior wall aneurysm 20% of area
Tsui et al., 2001	34	None	Atonic uterus after cesarean section for failure to progress	250‐mcg IV ergometrine once only	Unresponsive and bradycardic with progression to asystolic cardiac arrest and VF. Resuscitated	CK 2763 U/L	Acute anterior infarct with inferior ST depression	Diffuse spasm LAD and left circumflex, subtotal occlusion in principal diagonal branch of LAD. LVEF 15%	200‐mcg intracoronary nitroglycerin	Intra‐aortic balloon pump, inotropes. Further inferoposterior ischemic ECG changes reversed with IV nitroglycerin. Discharged day 11
Hayashi et al., 2003	25	FHx mother (angina) Prior history of occasional “chest oppression at rest”	Postpartum bleeding	200‐mcg IV methylergometrine once only	“Chest oppression, palpitation, and nausea”	CK 928 U/L (MB fraction 66 U/L)	ST‐segment depression in precordial leads		IV nitrates	Cardiac catheterization after 3 mo—normal coronary arteries, LV hypokinesis, LVEF 58%. Nil coronary artery spasm with IV ergometrine Long‐term diltiazem Subsequent delivery avoided ergometrine
Kuczkowsi, 2004	36	None	Elective repeat CS for fetal macrosomia. Atonic uterus despite oxytocin and fundal massage	200‐mcg intramyometrial methylergometrine	“Almost immediate onset severe left‐sided substernal chest pain, radiating to her left arm, and shortness of breath”	CK negative	Nonspecific T‐wave abnormalities and transient ST‐segment elevation	Not documented	250‐mcg IV nitroglycerin	Nil evidence of myocardial ischemia or infarction on post‐op ECG or CK
Eom et al., 2005	40	None	Cesarean section, postpartum bleeding 1500mL	“One ampoule Erovin” (dose and route of administration not specified)	“Chest pain”	CK 162 U/L Trop negative	ST elevation II, III, aVF. ST depression V1, V2	Not performed	Nitroglycerin, IV fluids for hypovolemia	Cardiac arrest, CPR for 1 h with intubation, deceased. Autopsy: RCA, LAD, LCA, LCX severe atherosclerosis and calcification
Lin et al., 2005	38	Intermittent hypertension nil Rx	Termination of pregnancy at 5‐wk gestation. Routine prophylactic oxytocin and methylergometrine	200‐mcg IV methylergometrine once only	Chest pain, unresponsive, cardiac arrest	Not reported	Not reported	Not performed	Resuscitation	CPR for 70 min, deceased. Autopsy: nil evidence stroke, thromboembolism, atherosclerosis, aortic dissection, LVH
de Labriolle et al., 2009	38	Smoking 12 pack‐years	Termination of pregnancy with methylergometrine. Onset of symptoms after 3 d of therapy, resolving spontaneously then recurring day 4 after continued therapy	125‐mcg PO methylergometrine TDS for 3 d	Retrosternal chest pain irradiating to both arms and neck	Trop I 34.6 mcg/L, CK 1555 mg/L	Subepicardial ischemia V2, V3, V4 and D1VL, Q waves V2, V3	After 2 d: nil coronary abnormalities. After 6 d: provocation 400‐mcg methylergometrine produced narrowing 2nd segment LAD	Sublingual nitrates (ineffective)	Echocardiography: apical akinesia, LVEF 48%
Santoro et al., 2012	44	Smoker	Oral methylergometrine for gynecologic bleeding	425‐mcg PO methylergometrine daily for 0.5 wks	Chest pain, nausea, and vomiting	Trop I 0.56 ng/mL	ST‐segment elevation anterior and lateral leads with ventricular tachycardia. Echo: LVEF 30% and akinesis of apex, anterior and lateral walls	Mild stenosis mid‐LAD	Coronary stenting	LVEF > 55%, T‐negative waves anterior leads
Ramzy et al., 2015	36	Essential hypertension managed with atenolol	Incomplete miscarriage 6‐wk gestation. Dilatation and curettage with prophylactic ergometrine	500‐mcg IV ergometrine once only	Intraoperative cardiac monitoring	Trop hs‐Tnt 5504 ng/L, CK 1962 U/L	Anteroseptal ST elevation	Echocardiogram: anteroseptal hypokinesis. Angiography after 1 d: normal coronary arteries	Low‐dose noradrenaline for BP support, aspirin, IV heparin	Discharged on aspirin. Atenolol recommenced 2 wks. 2‐mo review: asymptomatic, normal LV function on TTE

Note

Normal ranges (as per Royal College of Pathologists of Australia)^30^—creatinine kinase 30‐180 U/L (adult female); troponin I/troponin T not detectable for conventional assays and point‐of‐care testing/assay and population dependent for high‐sensitivity troponin assays (99th %)

Abbreviations: AMI, acute myocardial infarction; BP, blood pressure; CK, creatinine kinase; CPR, cardiopulmonary resuscitation; CS, cesarean section; ECG, electrocardiogram; FHx, family history; GTN, glyceryl trinitrate; hs‐Tnt, high‐sensitivity troponin T; IM, intramuscular; IU, international units; IV, intravenous; L, liter; LAD, left anterior descending; LCA, left coronary artery; LCX, left circumflex; LV, left ventricle; LVEF, left ventricular ejection fraction; LVH, left ventricular hypertrophy; mcg, micrograms; mg, milligrams; MI, myocardial infarction; mL, milliliter; mV, millivolts; ng, nanogram; PO, per os (oral); PPH, postpartum hemorrhage; RCA, right coronary artery; rtPA, recombinant tissue plasminogen activator; SL, sublingual; TDS, ter die sumendum (three times per day); Trop I, troponin I; Trop, troponin; U/L, units per liter.

In these two cases, administration of ergometrine in the context of significant anemia precipitated myocardial ischemia. Correction of the anemia and supportive therapy while the ergometrine was metabolized were sufficient to reverse the myocardial ischemia, and these patients did not require therapeutic anticoagulation or invasive cardiac treatment and were free of long‐term complications. A review of the literature reminds us that myocardial ischemia can result in pathological changes to the cardiac anatomy such as wall hypokinesis and aneurysm formation,^2^, ^6^, ^7^, ^9^, ^13^, ^14^, ^26^, ^28^ exacerbation of coronary artery disease,^2^, ^3^, ^4^, ^7^, ^8^, ^27^, ^28^, ^29^ and death.^4^, ^5^

## 4 CONCLUSIONS

Ergometrine is commonly administered in obstetrics for management of postpartum hemorrhage secondary to uterine atony. In these two cases, close attention to the patients’ symptoms, appropriate cardiac monitoring, and postdelivery cardiac assessment ensured timely recognition and subsequent management. Short‐term follow‐up demonstrated no apparent impact on cardiac function although long‐term follow‐up of such patients has not been described.

Bateman et al^10^ suggest that the administration of an ergot in the peripartum period does not significantly increase the risk of acute coronary syndrome or acute myocardial infarction, but these cases highlight the continued importance of recognizing the rare occurrence of myocardial ischemia induced by ergometrine in the context of the anemic state induced by postpartum hemorrhage.

## CONFLICT OF INTEREST

The authors declare that they have no competing interests.

## AUTHORS’ CONTRIBUTIONS

SL: identified the cases and critically revised the manuscript for important intellectual content. SS: was the major contributor in collecting the case information, conducting the literature review, and writing the manuscript. All authors read and approved the final manuscript.

## ETHICAL APPROVAL

Not applicable.

## CONSENT FOR PUBLICATION

Written informed consent was obtained from both patients for publication of these case reports. Copies of the written consents are available for review by the editor in chief of this journal.

## Data Availability

Not applicable.
